# Correlation of PD-L1 expression with different clinico-pathological and immunohistochemical features of ovarian surface epithelial tumors

**DOI:** 10.1007/s12094-024-03613-2

**Published:** 2024-08-01

**Authors:** Asem Shalaby, Ola Shalaby, Hazem Abdullah, Mohamed Rachid Boulassel, Mohammad Arafa

**Affiliations:** 1 Pathology Department, Faculty of Medicine,, Mansoura University, Mansoura, Egypt; 2 Pathology Department, College of Medicine and Health Sciences, Sultan Qaboos University, Muscat, Oman; 3 Pathology Department, Faculty of Medicine, Damietta University, Damietta, Egypt; 4 Department of Hematology, College of Medicine and Health Sciences, Sultan Qaboos University, Muscat, Oman; 5 Department of Biomedical Science, College of Medicine and Health Sciences, Sultan Qaboos University, Muscat, Oman

**Keywords:** PD-L1, Carcinoma, ER, PR, P53

## Abstract

**Background:**

Primary carcinoma of the ovary (OCs) are responsible for a significant number of deaths related to cancer, and have the highest rate of death related to cancers of the female reproductive organs. Programmed cell death 1 (PD1) protein, acts as an immune checkpoint, and has an important role in the down-regulation of the immune system by preventing the activation of T-cells, which will weaken the autoimmunity and increases self-tolerance. This study aimed at the evaluation of the immunohistochemical (IHC) expression of PD-L1 in various primary surface ovarian epithelial tumours and to test its correlation with different clinicopathological parameters together with the expression of a panel of P53, ER and PR.

**Methods:**

A set of 102 cases of primary ovarian surface epithelial neoplasms (benign, borderline and malignant) were collected to construct Tissue Microarray (TMA) using 3 tissue cores from each case. IHC for PD-L1, p53, PR and ER was performed. The expression of PD-L1 was evaluated in relation to some clinicopathological parameters and to the expression patterns of other markers.

**Results:**

Expression of PD-L1 was detected in about 51% (*n* = 36) of malignant tumours. The malignant group significantly showed PD-L1 positivity compared to borderline and benign groups. The malignant tumours significantly showed PD-L1 and total p53 positivity in comparison to borderline group. Also, malignant tumours significantly showed higher combined positivity of PD-L1 and either PR or ER compared to borderline and benign lesions. No significant correlation was appreciated between PD-L1 expression and with any of the studied clinicopathological parameters.

**Conclusions:**

This study showed a significant PD-L1 expression in malignant primary surface epithelial tumours. Construction of a panel of IHC markers, including PD-L1, could have a potential value to define patients those would benefit from the addition of immunotherapy to the treatment plan.

## Background

Ovarian primary malignant epithelial neoplasms, known as ovarian carcinomas (OCs), constitute a significant fraction of deaths related to cancer in women and has the highest cancer related death rate amongst cancers of the female reproductive organs. The disease is lethal particularly due to late initial presentation and the lack of early diagnosis due to its vague symptoms [1, 2]. Traditionally, ovarian tumours can be grouped into two distinct types [2, 3]. Type I represents about 30% of ovarian cancers. It is genetically stable, slowly growing, and comprises low-grade serous, endometrioid, clear-cell, and mucinous carcinomas. On the other hand, type II accounts for about 70% of cases and is more aggressive, genetically unstable. This includes high-grade serous carcinomas and carcinosarcomas. This classification can shed light on the capacity of tumoral cells to escape immune clearance, resulting in the tumour cell propagation [3].

Despite advances in treatment modalities, the overall cure rate has remained around 30% for ovarian cancers. Such poor outcome could be explained by the delay in initial diagnosis so that many cases present initially at an advanced stage. This highlighted the need for other therapeutic modalities and personal tailored management [1, 2].

Nowadays, it has been shown that the inhibitors of PD1 or PD-L1 could reduce tumor proliferation and, therefore, can be used in the treatment of many tumours. This led to an expanded use of inhibitors of PD-L1 such as Pembrolizumab and Nivolumab and selection of patient candidates for immunotherapy [4].

Programmed cell death 1 (PD1) shows surface expression for different immune cells, including T lymphocytes. PD1 becomes active by one of its ligands, PD-L1 or PD-L2, and expressed by antigen-presenting cells such as macrophages and B lymphocytes [5]. PD-L1 might be involved in the process of increase in size and progression of the tumour, and therefore, leading to poor prognosis [6].

PD-L1 expression might reflect an aggressive potential of the tumour and could play a significant role in tumour immune escape [4].

Although PD-L1 is considered to be a strong negative regulator of anti-tumour immune response, little is known about the way membranous and cytoplasmic. Membranous expression of PD-L1 are regulated by the tumour environment in carcinoma of the ovaries [5]. It was noted that PD-L1 and PD-L2 were highly expressed in ovarian carcinomas. The levels of expression of PD-L1 and PD-L2 were correlated with age, FIGO stage, and prognosis, results suggesting their roles in the initiation and development of malignant ovarian tumours [7]. On the other hand, the expression of PD-L1 was reported not to be associated with patient risk for ovarian cancer [8]. Many clinical trials utilizing checkpoint inhibitors in ovarian cancer patients concluded that the expression of PD-L1 in ovarian cancer cells, the histotype, and previous treatment are associated with the success of immune therapies. However, and until recently, the literature is not sufficient to allow for the identification and selection of ovarian cancer patients who would successfully respond to immunotherapy [3, 9].

It has been found that elevation of PD-L1 levels is because of decreased level of miR-200 resulting in disturbed function of CD8 T-cells, which is coupled with epithelial-mesenchymal transition (EMT) to enhance metastasis [8]. PD-L1 and EMT could regulate each other to form feed-forward regulation [7]. Similar to any treatment modalities, immunotherapy has some reported disadvantages. For example, immunotherapy resistance mechanisms might occur in the form of primary resistance, adaptive immune resistance, or acquired resistance. Furthermore, it was reported that some of ovarian cancer patients experienced early treatment discontinuation due to radiographic or clinical disease progression [10, 11]. According to the WHO classification of ovarian tumours, surface epithelial tumors of the ovary are categorized into various subtypes including serous, mucinous and endometrioid tumours [3]. To achieve an accurate diagnosis, immunohistochemistry is widely used in typing and prognostication of ovarian tumors. For example, p53 gene immunohistochemical expression patterns can be used to differentiate high grade serous carcinoma (HGSC) in which the expression is diffuse and intense (mutant) from low-grade serous carcinoma (LGSC) that usually show (wild-type) p53 staining, with a weak focal, and heterogeneous [12]. Moreover, the analysis of steroid receptors ER/PR in ovarian carcinoma showed significant favorable prognostic values. It was reported that progesterone receptor positivity is proved to be an independent prognostic variable of improved progression for free-survival among patients with ovarian cancers [13].

In the current study, we aimed to assess the immunohistochemical expression of PD-L1 in different ovarian tumor cases. Also, to study PD-L1 expression in relation to clinicopathological parameters. Finally, to study the correlation between PD-L1 and a panel of p53, PR, PR in relation to each other.

## Materials and methods

### Sample selection

The study was performed utilising the data of ovarian surface epithelial tumor patients received in the Pathology Department, Oncology Centre, Mansoura University Hospitals in the period from 2010 to 2014. A total number of 102 cases of surface epithelial tumours (benign, borderline and malignant) were included in the analysis. The diagnostic categories were as follow; serous tumors were represented as 49 carcinoma cases, nine borderline and six benign cases, whereas mucinous tumors accounted for eleven carcinoma cases, five borderline and ten benign cases. There were five endometrioid carcinomas, four undifferentiated carcinomas. There were one case of clear cell carcinoma and two cases of benign Brenner tumor. The overall parentage of the lesions was 68.3% (70 cases) malignant, 13.8% (14 cases) borderline and 17.8% (18 cases) benign. All data of the patients in the medical records were used as inclusion criteria for the study. The cases were re-evaluated and confirmed for histological type, grade, and stage.

All the procedures were approved by the Ethics Committee of the Mansoura University [Institutional Review Board (IRB) Ref. MDP.19.10.31 dated October 2019] and in agreement with Declaration of Helsinki of 1964 and its later amendments. There were no required written consents with a waiver from the Institutional Review Board.

### Construction of tissue microarray (TMA)

Tissue microarray (TMA) was constructed as described previously [14, 15]. Briefly, after reviewing the routine H&E-stained slides, representative tumour slides were selected, and the tumour area on the selected slide, per case, was encircled. The corresponding paraffin blocks were, retrieved. A manual tissue arrayer (Beecher Instruments Inc., Sun Prairie, WI, USA) was used and the selected areas from the donor blocks were cored with a 0.6-mm-diameter cylinder tissue punch and placed in the recipient blocks. Triplicate cores from every case were taken from the tumour.

### Immunohistochemistry

Tissue microarray sections were obtained at a thickness of 4 µm and stained immunohistochemically by an automated stainer (BenchMark, Roche, Tucson, AZ, USA), with antibodies against PD-L1 clone 22C3 (Dako, Carpenteria, CA, USA), P53 (clone DO7, Ventana, Tucson, AZ), ER (clone SP1, Ventana, Tucson, AZ), PR (clone 1E2, Ventana, Tucson, AZ). The procedures were performed according to the manufacturers’ instructions.

### Evaluation of immunohistochemical reactivity

The stained slides were evaluated by two pathologists (AS, MA), with no prior knowledge of the patient details and outcomes, using standard light microscopes.

### PD-L1

According to the intensity of the stain, PD-L1 expression was categorized into four groups, as follows: 0 (negative expression), 1 + (positive expression but weaker than placenta), 2 + (equivalent to expression in placenta), and 3 + (stronger expression than in placenta). Positive staining of the non-neoplastic areas and the immune cells were excluded. Expression of PD-L1 ≥ 2 + was considered as positive [16].

### P53, ER and PR

A semi-quantitative score was used considering the average proportion of positive cells plus average intensity. The immunoreactive cells were scored as score 0 (0%), score 1 (1–25%), score 2 (26–50%), score 3 (51–75%) and score 4 (> 75%). The average intensity of the immunoreactivity was scored as score 0 (negative), score 1 (weak), score 2 (moderate) and score 3 (intense).

For P53, the final scores were divided into three different patterns; the null “negative” pattern (score 0): no reactive nuclei or reactivity in < 5% of nuclei, the wild-type pattern (scores 2–6): weak to moderate reactivity in 5–75% and the overexpressed pattern (score 7): strong nuclear reactivity in > 75%. For malignant lesions, null and overexpressed phenotypes refer to mutant p53 status [17]. For PR and ER, scores > 2 considered positive and scores > 5 considered strong positive [18].

### Statistical analysis

SPSS Version 22.0 (IBM Corp., Armonk, NY, USA) was used to analyse the data. Numbers and percentages were used to describe and express qualitative data. When describing quantitative data that were not parametric, the median (range) was used. After utilizing the Kolmogrov–Smirnov test to verify normalcy, the mean [standard deviation (SD)] was utilized for parametric data and for non-parametric data. The results’ significance was assessed at the 0.05 level. The significance of the differences in categorical variables between the groups was examined using the chi-squared test. When more than 25% of cells in tables (more than 2 × 2) had a count of less than 5, the Monte Carlo test was used as a correction for the Chi-squared test. Independent non-normally distributed data were compared using the Mann–Whitney U test. When comparing continuous, non-normally distributed continuous data, Spearman’s correlation was employed. In order to determine how risk factors affected survival, the Kaplan–Meier test was utilized to compute both overall survival and disease-free survival using the log rank test.

## Results

In the current study, a total of 102 patients with different types of ovarian surface epithelial tumours were included in the analysis. The patients’ mean age was 50, ranged from 17 to 79 years old.

### PDL-1 expression

This showed that 51.4% of malignant group have positive PDL-1 expression, however 48.6% were negative. The majority of borderline group 71.4% showed positive PDL-1. On the contrary, the majority of benign group 67.7% showed negative PDL-1 expression. The malignant group significantly showed more PD-L1 positivity compared to borderline and benign groups. The results of expression of PD-L1, ER, PR and P53 in different categories of ovarian tumors (benign, borderline and malignant) are shown in Table 1.

**Table 1:** Expression of ER, PR, p53 and PD-L1 in the malignant, borderline and benign groups

Tumor markers	Histopathological group		
	Malignant (*n* = 70)	Borderline (*n* = 14)	Benign (*n* = 18)
Protein 53 [*n*, (%)]			
Mutant*	57 (81.4)	8 (57.1)	18 (100)
Wild type	13 (18.6)	6 (42.9)	0 (0.0%)
PR score [*n*, (%)]			
Positive	19 (27.2)	7 (50.0)	3 (16.7)
Negative	51 (72.8)	7 (50.0)	15 (83.3)
ER score [*n*, (%)]			
Positive	12 (17.2)	6 (42.9)	4 (22.2)
Negative	58 (82.8)	8 (57.1)	14 (77.8)
PDL-1 score [*n*, (%)]			
Positive	36 (51.4)	10 (71.4)	6 (33.3)
Negative	34 (48.6)	4 (28.6)	12 (67.7)

### Expression of ER, PR, p53 and PD-L1 in the malignant, borderline and benign groups

PDL-1 expression was studied in relation to the characteristics of the malignant group. There were non-significant relation to its expression and all characteristics including the malignant type, tumor grade, size, laterality, age and presence or absence of metastasis (Table 2).

**Table 2:** Relation of PDL-1 positivity to different clinico-pathological parameters of malignant tumours

Characteristics	Positive *n* (%)	Negative *n* (%)	*P* values
Malignant type (*n* = 70)	36 (51.4)	34 (48.6)	0.81
High grade (*n* = 37) (High-grade serous and undifferentiated carcinomas)	18 (48.6)	19 (51.4)	0.86
Low-grade carcinomas (*n* = 33) (Low-grade serous, mucinous, endometrioid and clear cell)	18 (54.5)	15 (45.4)	0.60
Metastasis present (*n* = 53)	28 (52.8)	25 (47.2)	0.68
Metastases absent (*n* = 17)	8 (47.0)	9 (53.0)	0.80
Unilateral site (*n* = 29)	15 (51.7)	14 (48.3)	0.85
Bilateral site (*n* = 41)	21 (51.2)	20 (48.8)	0.87
Age < 55 years (*n* = 34)	17 (50)	17 (50)	1.0
Age > 55 years (*n* = 36)	19 (52.7)	17 (47.2)	0.73
Tumor size ≤ 11 cm (*n* = 29)	19 (65.5)	10 (34.5)	0.35
Tumor size > 11 cm (*n* = 41)	19 (46.3)	21 (53.7)	0.63

There was an evident significance in combining the expression of PDL-1 with the other studied markers (p53, ER and PR) in the malignant group as shown in Table 3. The study showed that 92.9% of the malignant group showed triple negative panel of PDL-1, ER and PR compared to 7.1% showing triple positivity (*p* < 0.0001).

**Table 3:** Relation of PD-L1positivity to ER, PR and p53 reactivity

Characteristics	Markers	Positive (*n*, %)	*P* values
Malignant group (*n* = 70)	PD-L1 alone	36 (51.4)	0.81
	PD-L1 + ER	8 (11.4)	0.0001
	PD-L1 + PR	14 (20)	0.0001
	PD-L1 + P53 heterogeneous	6 (8.6)	0.0001
	PD-L1 + P53 mutated	20 (28.6)	0.0001
	PD-L1 + ER + PR	5 (7.1)	0.0001

Furthermore, the malignant group significantly showed a higher PD-L1 and total P53 positivity compared to borderline group. Also, the malignant group showed significantly PD-L1 and either PR or ER positivity compared to the borderline and benign groups.

**Fig. 1:**
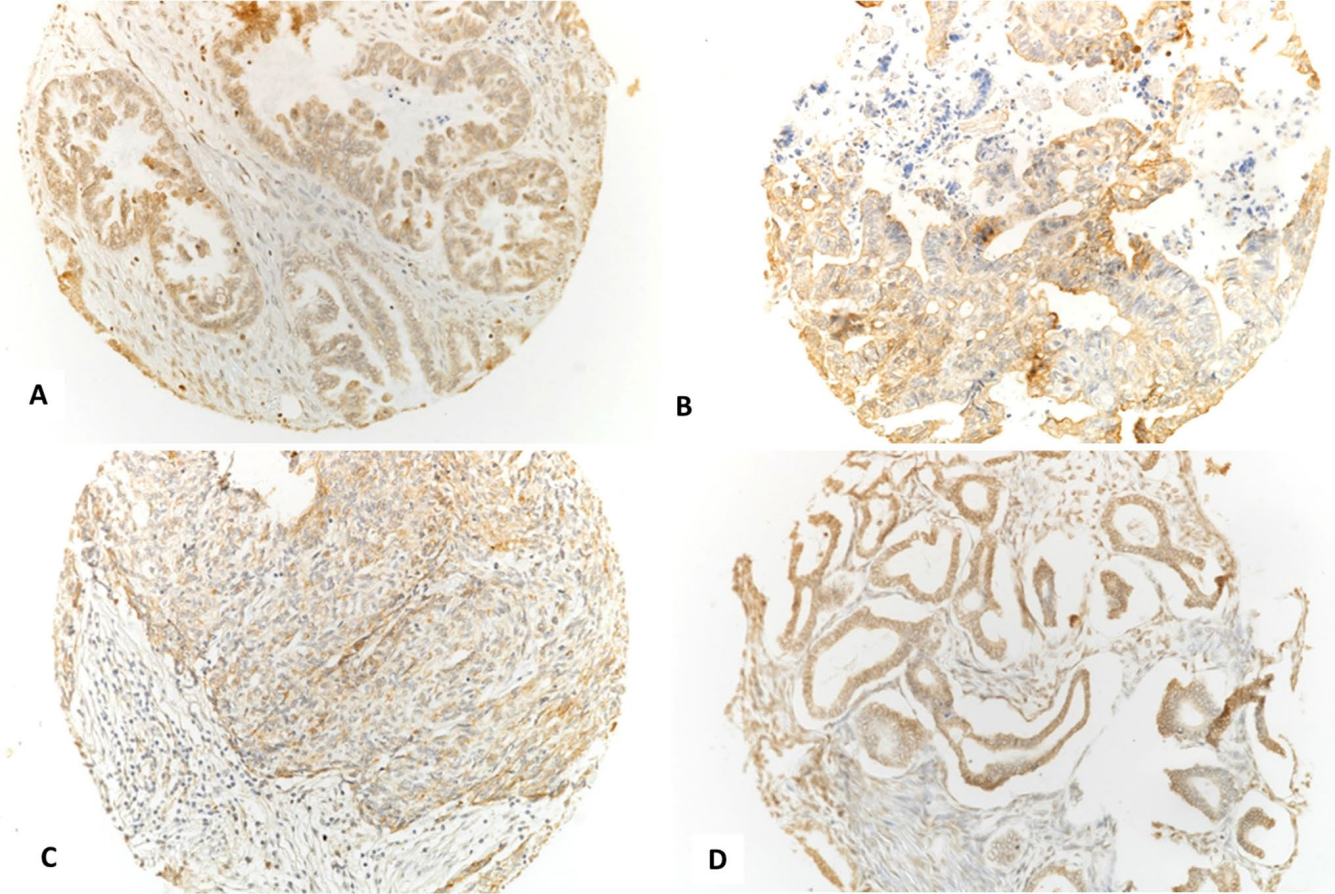
Photomicrographs showing examples of PD-L1 immunohistochemical expression as cytoplasmic staining in different entities of surface epithelial ovarian tumours; borderline serous tumour (**A**), low-grade serous carcinoma (**B**), high-grade serous carcinoma (**C**) and mucinous carcinoma (**D**)

Examples of the different immunohistochemical expression patterns of the studied markers are shown in Fig. 1.

## Discussion

As an immunological checkpoint, programmed cell death protein 1 (PD1) is crucial in suppressing the immune system by preventing T-cell activation, which reduces autoimmunity and fosters self-tolerance [4].

As of right now, it has been shown that immune checkpoint inhibitors that target PD1 or PD-L1 have the potential to slow tumor growth and be applied to the treatment of a number of cancer types.3. The usage of PD-L1 inhibitors, such as pembrolizumab and nivolumab, has rapidly expanded due to recent advancements in immunotherapies and the classification of several tumor types as immunogenic tumors [19].

The current investigation found that, with variations based on histological subtypes, a comparatively small number of OCs express PD-L1, a finding that is broadly consistent with earlier findings [20]. The number of high-grade serous carcinomas that expressed PD-L1 in that study was over 40%, which is less than what we have found. Variability in the methods, such as the monoclonal antibody utilized, may account for this [20]

Furthermore, a meta-analysis showed that the percentage of ovarian cancers expressing PD-L1 varied widely (11% to 88%). Differences in the scoring system, the antibodies utilized, and the histological subtypes could potentially account for these apparent variations [21].

Our findings are consistent with earlier research showing a higher correlation between PDL-1 expression and malignant entities [22]. Additionally, our investigation showed that the malignant group had 51.4% positive expression of PD-L1, with no distinction between high-grade and low-grade tumors. This result is comparable to that of the Nhokaew et al., (2019) study, which found that elevated PD-L1 expression was present in the surgical specimens from approximately 63% of the cases. The rate of high expression of PD-L1 in surface epithelial ovarian carcinomas of type 1 or type 2 was not shown to differ significantly [16].

There is a severe lack of prior predictive evidence about PD-L1 expression in type I epithelial OCs in particular. A 2017 study by Zhu and colleagues analyzing data from 122 women with ovarian clear cell carcinoma showed that elevated PD-L1 expression levels were linked to advanced stages, recurrence, and worse prognosis [23]. In the current study, 8 instances had no metastases and 28 cases with metastases tested positive for PD-L1. This is consistent with research by Nhokaew and colleagues [16] that found that women with high PD-L1 expression were more likely than those with low marker expression to present with advanced stages of the disease and experience a recurrence of it. It has been shown previously that the expression of PD-L1 is relatively uncommon in epithelium ovarian tumors. In addition, the overall and progression-free survival between PD-L1 positive and PD-L1 negative patients were not different across all of the histological types, and each subtype in particular for serous carcinomas expressing PD-L1 [24].

Regarding the other markers individually, p53 IHC revealed that only 11.1% of cases of mucinous carcinoma had overexpressed p53, compared to 34.1% of cases of serous carcinoma, which showed overexpression in 45.5% of cases. This is consistent with other research, such as that conducted by Morita and associates, who found that p53 overexpression was more common in serous adenocarcinomas (63%) compared to mucinous adenocarcinomas (22%) [25, 26].

The current analysis showed a little variation from a previously published work [27] in that ER was expressed in cases of mucinous carcinomas (data not shown) but in slightly lower proportions than PR in endometroid carcinomas. Similar to earlier observations, PR was shown to be primarily expressed in endometroid carcinomas [28]. Contrary to the previously stated work [23], PR was not expressed in the CCC in our sample. Our extremely limited sample size may be the reason for the lack of substantial correlation seen between clinicopathological factors and hormone receptor expression [27, 28]. Moreover, the use of TMA method and the heterogeneity of tumor might hinder the accurate expression of the marker. However, the TMA technology was proved effective as being validated previously or the evaluation of PD-L1 [24].

## Conclusion

In conclusion, testing PDL-1 immunohistochemical expression in ovarian surface epithelial tumours might have significant impact on patients’ management. The present study shed light on the correlation between PDL-1 expression and a panel of immunohistochemical markers “p53, ER and PR” in a subset of surface ovarian tumors and its potential value in planning the treatment. As the malignant tumors showed significantly higher expression of PDL-1, either alone or in combination with the other markers, these group of patients might benefit from additional immunotherapy.

## Data Availability

Datasets used and/or analyzed during the current study are available from corresponding author upon reasonable request.

## Abbreviations

PD-L1: Programmed cell death ligand 1
OCs: Ovarian carcinomas
ER: Estrogen receptors
PR: Progesterone receptors
PD 1: Programmed cell death
IHC: Immunohistochemical

## References

[1] Cho KR, Shih IM. Ovarian cancer. Annu Rev Pathol. 2009;4:287–313. 10.1146/annurev.pathol.4.110807.092246. 18842102 10.1146/annurev.pathol.4.110807.092246 PMC2679364

[2] Cheung AN, Ellenson LK, Gillks CB, et al. editors. WHO Classification of Female Genital Tumors. 5th ed. Volume 70 WHO Classification of Tumors Editorial Board, International Agency for Research on Cancer (IARC); Lyon, France: 2020.

[3] Dumitru A, Dobrica EC, Croitoru A, Cretoiu SM, Gaspar BS. Focus on PD-1/PD-L1 as a therapeutic target in ovarian cancer. Int J Mol Sci. 2022;23(20):12067. 10.3390/ijms232012067. 36292922 10.3390/ijms232012067 PMC9603705

[4] Kahraman DS, Diniz G, Sayhan S, et al. The prognostic significance of pdl1 and foxp3 expressions in tumor cells and the tumor microenvironment of ovarian epithelial tumors. Int J Clin Exp Pathol. 2018;11(8):3884–90. 31949776 PMC6962806

[5] Que Y, Xiao W, Guan YX, et al. PD-L1 expression is associated with FOXP3+ regulatory T-cell infiltration of soft tissue sarcoma and poor patient prognosis. J Cancer. 2017;8(11):2018–25. 28819402 10.7150/jca.18683 PMC5559963

[6] Chen J, Jiang CC, Jin L, et al. A novel role of pro-survival signalling in cancer. Ann Oncol. 2016;27(3):409–16. 26681673 10.1093/annonc/mdv615

[7] Xue C, Zhu D, Chen L, et al. Expression and prognostic value of PD-L1 and PD-L2 in ovarian cancer. Transl Cancer Res. 2019;8(1):111–9. 35116740 10.21037/tcr.2019.01.09 PMC8797717

[8] Piao J, Lim HJ, Lee M. Prognostic value of programmed cell death ligand-1 expression in ovarian cancer: an updated meta-analysis. Obstet Gynecol Sci. 2020;63(3):346–56. 32489980 10.5468/ogs.2020.63.3.346 PMC7231937

[9] Garrido MP, Fredes AN, Lobos-González L, Valenzuela-Valderrama M, Vera DB, Romero C. Current treatments and new possible complementary therapies for epithelial ovarian cancer. Biomedicines. 2021;10(1):77. 10.3390/biomedicines10010077. 35052757 10.3390/biomedicines10010077 PMC8772950

[10] Yang C, Xia BR, Zhang ZC, Zhang YJ, Lou G, Jin WL. Immunotherapy for ovarian cancer: adjuvant, combination, and neoadjuvant. Front Immunol. 2020;11:577869. 10.3389/fimmu.2020.577869. 33123161 10.3389/fimmu.2020.577869 PMC7572849

[11] Alsuliman A, Colak D, Al-Harazi O, et al. Bidirectional crosstalk between PD-L1 expression and epithelial to mesenchymal transition: significance in claudin-low breast cancer cells. Mol Cancer. 2015;14:149. 26245467 10.1186/s12943-015-0421-2 PMC4527106

[12] McCluggage WG. Immunohistochemistry in the distinction between primary and metastatic ovarian mucinous neoplasms. J Clin Pathol. 2012;65(7):596–600. 21768188 10.1136/jcp.2010.085688

[13] Sharifi N, Yousefi Z, Saeed S, et al. Prognostic values of estrogen and progesterone expression receptors in ovarian papillary serous carcinoma. Iran J Pathol. 2009;4:9–12.

[14] Arafa M, Somja J, Dehan P, et al. Current concepts in the pathology and epigenetics of endometrial carcinoma. Pathology. 2010;42(7):613–7. 21080868 10.3109/00313025.2010.520307

[15] Salama A, Arafa M, ElZahaf E, et al. Potential role for a panel of immunohistochemical markers in the management of endometrial carcinoma. J Pathol Transl Med. 2019;53(3):164–72. 30813708 10.4132/jptm.2019.02.12 PMC6527935

[16] Nhokaew W, Kleebkaow P, Chaisuriya N, et al. Programmed death ligand 1 (PD-L1) expression in epithelial ovarian cancer: a comparison of type I and type II tumors. Asian Pac J Cancer Prev. 2019;20(4):1161–9. 31030490 10.31557/APJCP.2019.20.4.1161 PMC6948887

[17] Nafisi H, Ghorab Z, Ismill N, et al. Immunophenotypic analysis in early Müllerian serous carcinogenesis. Int J Gynecol Pathol. 2015;34(5):424–36. 10.1097/PGP.0000000000000179. 26107560 10.1097/PGP.0000000000000179

[18] Fadare O, James S, Desouki MM, et al. Coordinate patterns of estrogen receptor, progesterone receptor, and Wilms tumor 1 expression in the histopathologic distinction of ovarian from endometrial serous adenocarcinomas. Ann Diagn Pathol. 2013;17(5):430–3. 23706170 10.1016/j.anndiagpath.2013.04.011 PMC4079538

[19] Diniz G, Unlu I, Komurcuoglu B. Histopathologic and molecular features of lung cancers. Anatol J Med. 2017;27(2):77–87.

[20] Eymerit-Morin C, Ilenko A, Gaillard T, et al. PD-L1 expression with QR1 and E1L3N antibodies according to histological ovarian cancer subtype: a series of 232 cases. Eur J Histochem. 2021;65(1):3185. 33728864 10.4081/ejh.2021.3185 PMC7967270

[21] Huang LJ, Deng XF, Chang F, et al. Prognostic significance of programmed cell death ligand 1 expression in patients with ovarian carcinoma: a systematic review and meta-analysis. Medicine (Baltimore). 2018;97(43): e12858. 30412078 10.1097/MD.0000000000012858 PMC6221561

[22] Dumitru A, Dobrica EC, Croitoru A, et al. Focus on PD-1/PD-L1 as a Therapeutic Target in Ovarian Cancer. Int J Mol Sci. 2022;23(20):12067. 36292922 10.3390/ijms232012067 PMC9603705

[23] Zhu J, Wen H, Bi R, et al. Prognostic value of programmed death-ligand 1 (PD-L1) expression in ovarian clear cell carcinoma. J Gynecol Oncol. 2017;28(6): e77. 29027395 10.3802/jgo.2017.28.e77 PMC5641527

[24] Eymerit-Morin C, Ilenko A, Gaillard T, Varinot J, Compérat E, Bendifallah S, Darai E. PD-L1 expression with QR1 and E1L3N antibodies according to histological ovarian cancer subtype: a series of 232 cases. Eur J Histochem. 2021;65(1):3185. 10.4081/ejh.2021.3185. 33728864 10.4081/ejh.2021.3185 PMC7967270

[25] Morita K, Ono Y, Fukui H, et al. Incidence of P53 and K-ras alterations in ovarian mucinous and serous tumors. Pathol Int. 2000;50(3):219–23. 10792785 10.1046/j.1440-1827.2000.01028.x

[26] Zhang S, Dolgalev I, Zhang T, et al. Both fallopian tube and ovarian surface epithelium are cells-of-origin for high-grade serous ovarian carcinoma. Nat Commun. 2019;10(1):5367. 31772167 10.1038/s41467-019-13116-2 PMC6879755

[27] Shen F, Zhang X, Zhang Y, et al. Hormone receptors expression in ovarian cancer taking into account menopausal status: a retrospective study in Chinese population. Oncotarget. 2017;8(48):84019–27. 29137401 10.18632/oncotarget.20251 PMC5663573

[28] Sieh W, Köbel M, Longacre TA, et al. Hormone-receptor expression and ovarian cancer survival: an Ovarian Tumor Tissue Analysis consortium study. Lancet Oncol. 2013;14(9):853–62. 23845225 10.1016/S1470-2045(13)70253-5 PMC4006367

